# Simultaneous Determination of Albendazole and Its Three Metabolites in Pig and Poultry Muscle by Ultrahigh-Performance Liquid Chromatography-Fluorescence Detection

**DOI:** 10.3390/foods10102350

**Published:** 2021-10-02

**Authors:** Zhaoyuan He, Zhixiang Diao, Yawen Guo, Kaizhou Xie, Lan Chen, Chun Xue, Yang Lu, Jinyuan Chen, Tao Zhang

**Affiliations:** 1 College of Animal Science and Technology, Yangzhou University, Yangzhou 225009, China; MZ120191027@yzu.edu.cn (Z.H.); 18352764521@163.com (Z.D.); DX120200135@yzu.edu.cn (Y.G.); xuechun0727@163.com (C.X.); luyangbisheng@163.com (Y.L.); MZ120191044@yzu.edu.cn (J.C.); zhangt@yzu.edu.cn (T.Z.); 2 Joint International Research Laboratory of Agriculture & Agri-Product Safety, Yangzhou University, Yangzhou 225009, China; DZ120200026@yzu.edu.cn; 3 College of Veterinary Medicine, Yangzhou University, Yangzhou 225009, China

**Keywords:** ultrahigh-performance liquid chromatography-fluorescence detection (UPLC-FLD), albendazole (ABZ) and its three metabolites, pig muscle, poultry muscle

## Abstract

A fast, simple and efficient ultrahigh-performance liquid chromatography-fluorescence detection (UPLC-FLD) method for the determination of residues of albendazole (ABZ) and its three metabolites, albendazole sulfone (ABZ-SO_2_), albendazole sulfoxide (ABZ-SO), and albendazole-2-aminosulfone (ABZ-2NH_2_-SO_2_), in pig and poultry muscle (chicken, duck and goose) was established. The samples were extracted with ethyl acetate, and the extracts were further subjected to cleanup by utilizing a series of liquid–liquid extraction (LLE) steps. Then, extracts were purified by OASIS^®^ PRiME hydrophilic-lipophilic balance (HLB) solid-phase extraction (SPE) cartridges (60 mg/3 mL). The target compounds were separated on an ACQUITY UPLC^®^ BEH C_18_ (2.1 mm × 100 mm, 1.7 μm) chromatographic column, using a mobile phase composed of 31% acetonitrile and 69% aqueous solution (containing 0.2% formic acid and 0.05% triethylamine). The limits of detection (LODs) and limits of quantification (LOQs) of the four target compounds in pig and poultry muscle were 0.2–3.8 µg/kg and 1.0–10.9 µg/kg, respectively. The recoveries were all above 80.37% when the muscle samples were spiked with the four target compounds at the LOQ, 0.5 maximum residue limit (MRL), 1.0 MRL, and 2.0 MRL levels. The intraday relative standard deviations (RSDs) were less than 5.11%, and the interday RSDs were less than 6.29%.

## 1. Introduction

**Figure 1:**
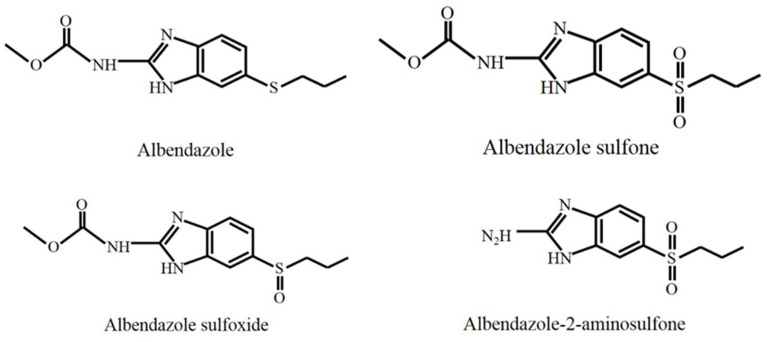
Structural formulas of ABZ, ABZ-SO_2_, ABZ-SO, and ABZ-2NH_2_-SO_2_.

Albendazole (ABZ) is a benzimidazole drug and a broad-spectrum, highly effective anthelmintic drug widely used in veterinary clinics. ABZ is metabolized into its major active metabolites albendazole sulfone (ABZ-SO_2_), albendazole sulfoxide (ABZ-SO) and albendazole-2-aminosulfone (ABZ-2NH_2_-SO_2_) in animals. The chemical structures of ABZ, ABZ-SO_2_, ABZ-SO, and ABZ-2NH_2_-SO_2_ are shown in Figure 1.

With increasing large-scale breeding, various parasitic diseases are seriously hindering economic output in the animal husbandry industry [1]. Many kinds of parasites infect animals, and parasites can parasitize various organs and tissues. ABZ has a good effect on gastrointestinal nematodes, lung nematodes, worms, and ectoparasites in pigs [2,3]. This drug is especially effective against *Ascaridia galli* and other worms in chickens [4]. Therefore, in many countries, ABZ is often used to treat these infections. However, some breeders do not use these drugs according to regulations, and abuse them in pursuit of greater economic benefits. These behaviors cause large amounts of these drugs to remain in animal-derived foods, such as muscle, fat, and internal organs, which can endanger the health of consumers [5]. In addition, animal toxicological studies have shown that ABZ and its metabolites may cause malformations and embryonic lethality [6,7]. Therefore, it is necessary to develop efficient and rapid detection methods for residues of ABZ and its three metabolites in animal products, to avoid harming consumer health. Most countries have stipulated the maximum residue limit (MRL) of ABZ in animal products [8,9]. The Ministry of Agriculture and Rural Affairs of the People’s Republic of China, the Codex Alimentarius Commission (CAC), and the European Union (EU) have stipulated that the MRL of ABZ in the muscles of all edible animals should not exceed 100 μg/kg, and the limit of the ABZ residue is based on the sum of ABZ and its three metabolites. This study adopted an MRL of 100 μg/kg in animal muscle as the standard, and developed a simultaneous detection method of ABZ and its three metabolites in pig and poultry muscle.

Many quantitative methods, such as high-performance liquid chromatography-ultraviolet detection (HPLC-UV) [10,11], high-performance liquid chromatography-fluorescence detection (HPLC-FLD) [12,13,14,15,16], and liquid chromatography-mass spectrometry (LC-MS) [17,18,19,20,21], have been established for the simultaneous detection of ABZ and its metabolites in food-producing animals. Among these methods, the most widely used has been LC-MS because of its high sensitivity and accuracy. However, LC-MS instruments are very expensive, causing the detection cost to be relatively high, and high-purity reagents and highly trained operators are strictly required. The HPLC-FLD method is time-consuming and has low detection efficiency, and some HPLC-FLD studies could not quantify all metabolites of ABZ. The detection process was very difficult and complicated when gas chromatography-mass spectrometry methods were used to determine ABZ and its metabolites, due to the basic nature and low volatility of these substances. The sensitivity of HPLC-UV was also not high enough [17]. It is very important to develop accurate, stable, and low-cost detection methods. At present, to our knowledge, there is no report on the simultaneous detection of ABZ and its three metabolites in pig or poultry muscle by ultrahigh-performance liquid chromatography-fluorescence detection (UPLC-FLD). Therefore, this study intended to develop a rapid, easy, and reliable UPLC-FLD method using liquid–liquid extraction (LLE) combined with solid-phase extraction (SPE) technology as a sample preparation technique to establish simultaneous detection of ABZ and its three metabolite residues in pig and poultry muscle (chicken, duck, goose).

## 2. Materials and Methods

### 2.1. Standards and Reagents

ABZ (CAS No. 54965–21–8, purity ≥ 98.0%), ABZ-SO_2_ (CAS No. 75184–71–3, purity ≥ 99.0%), and ABZ-SO (CAS No. 54029–12–8, purity ≥ 98.0%) were purchased from Sigma-Aldrich (MO, USA). ABZ-2NH_2_-SO_2_ (CAS No. 80983–34–2, purity ≥ 99.8%) was obtained from TMstandard (Changzhou, China).

HPLC-grade formic acid (purity ≥ 98%), trimethylamine (purity ≥ 99%), methanol (purity ≥ 99.9%) and acetonitrile (purity ≥ 99.9%) were purchased from Tokyo Chemical Industry (Tokyo, Japan), Thermo Fisher Scientific (MA, USA), and Merck (Darmstadt, Germany). Analytical-grade ethyl acetate, n-hexane and ammonia were supplied by Sinopharm Chemical Reagent Co. (Shanghai, China). The experimental water was ultrapure water (the resistivity reached 18.2 MΩ × cm, 25 °C).

### 2.2. Equipment

An ACQUITY UPLC System (Waters Corp, Milford, MA, USA) coupled to a fluorescence detector (Waters Corp, Milford, MA, USA) was used. Data acquisitions were performed by Empower 3 software.

Other equipment, such as an EnSpire multimode plate reader (PerkinElmer, Waltham, MA, USA), P300H-type ultrasonic cleaner (Elma, Munich, Germany), Vortex-Genie 2 vortex oscillator (Scientific Industries, MA, USA), N-Evap 112 nitrogen blower (Organomation, Columbus, OH, USA), 5810R high-speed refrigerated centrifuge (Eppendof, Hamburg, Germany), JJ-2/FK-A tissue homogenizer (Jiangsu Kexi Instrument Co., Ltd., Jiangsu, China), and AX205 analytical balance (Mettler-Toledo, Zurich, Switzerland), were also used in this study.

### 2.3. Standard Solutions

Stock standard solutions of ABZ, ABZ-SO_2_, ABZ-SO and ABZ-2NH_2_-SO_2_ were prepared by dissolving 2.5 mg of individual analytes in 25 mL of methanol to obtain a final concentration of 100.0 μg/mL. The stock standard solutions were stored stably for 3 months at −70 °C in the dark. The solutions required shaking for the analytes to fully dissolve, and sonication was applied to assist ABZ dissolution.

Working standard solutions (10.0, 1.0, and 0.1 μg/mL) of ABZ, ABZ-SO_2_, ABZ-SO and ABZ-2NH_2_-SO_2_ were prepared by diluting the stock standard solution (100.0 μg/mL) of each compound with acetonitrile and 0.2% formic acid aqueous solution containing 0.05% triethylamine (31:69, *v*/*v*). The working standard solutions were stored stably for 3 months at −34 °C in the dark.

### 2.4. Blank Samples and Sample Preparation

This study was conducted in accordance with the ethics requirements of the official ethical committee of our university. Muscle samples were obtained from chickens, ducks, geese, and a pig, none of which had received any medication. The samples were stored at −34 °C in a refrigerator after they were homogenized with a tissue homogenizer.

Two grams of homogeneous blank muscle were accurately weighed in 50 mL polypropylene centrifuge tubes after thawing. Samples were spiked with 15 mL of ethyl acetate, vortexed for 5 min by a vortex oscillator, ultrasonically extracted for 5 min, and centrifuged for 10 min at 12,000 rpm (4 °C). The supernatant was transferred into a new centrifuge tube after centrifugation. Subsequently, the precipitate was again extracted as before, and the supernatant was combined with that from the first extraction. The supernatants were blown to near dryness with nitrogen. The residue sample was dissolved in 5 mL of mobile phase and spiked with 15 mL of n-hexane saturated with acetonitrile for degreasing. Then, the sample was vortexed for 2 min and centrifuged for 5 min at 6000 rpm (4 °C). Subsequently, the n-hexane layer was discarded, and the supernatant was collected. After OASIS^®^ PRiME HLB SPE cartridges (60 mg/3 mL, Waters Corp, USA) were conditioned by 3 mL of methanol and 3 mL of ultrapure water, the supernatant was purified by OASIS^®^ PRiME HLB SPE cartridges. Then, the samples were eluted sequentially with 3 mL of mobile phase and 3 mL of 20% ammoniated methanol (ammonia water:methanol = 2:8, *v*/*v*), and the eluate solution was collected into 10 mL centrifuge tubes. The eluate solution was blown to near dryness with nitrogen. The residue sample was reconstituted with 2.0 mL of mobile phase, and the mixture was vortexed at low speed for 1 min. Finally, the samples were passed through a hydrophilic PTFE type (13 mm × 0.22 μm) needle (Thermo Fisher Scientific, USA), and the filtrate was analyzed by UPLC-FLD.

### 2.5. UPLC-FLD Analysis

A UPLC system equipped with a fluorescence detector was employed. Compound separation was executed on an ACQUITY UPLC^®^ BEH C_18_ (2.1 mm × 100 mm, 1.7 μm) chromatographic column (Waters Corp, USA) connected to a VanGuard^TM^ BEH C18 (2.1 mm × 5 mm, 1.7 μm) guard column (Waters Corp, USA) with an appropriate column temperature of 35 °C. Mobile phase A was acetonitrile, and mobile phase B consisted of 0.2% formic acid aqueous solution containing 0.05% trimethylamine. Mobile phases A and B were degassed quickly for 20 min by an ultrasonic cleaner before they were used. Isocratic elution was utilized in the method, and the ratio of mobile phases A and B was 31:69 (*v*/*v*). The flow rate was 0.25 mL/min, and the injection volume was 5 µL. The total run time was 6 min. The excitation wavelength and emission wavelength of the four compounds were 286 and 335 nm, respectively.

### 2.6. Method Validation

The procedure of the UPLC-FLD method was validated by referring to the requirements of the EU [22]. The validation criteria involved sensitivity, linearity, recovery and precision.

#### 2.6.1. Sensitivity

The sensitivity of the method was assessed in terms of the LODs and LOQs. When the signal-to-noise ratio (S/N) ≥ 3, the corresponding additive concentration was the LOD of the analytical method. When S/N ≥ 10, the corresponding additive concentration was the LOQ of the analytical method; at the same time, the concentration met the accuracy and precision requirements (recovery ≥ 70%, relative standard deviation (RSD) ≤ 20%). The working standard solutions of ABZ, ABZ-SO_2_, ABZ-SO and ABZ-2NH_2_-SO_2_ were diluted stepwise with each blank muscle matrix extract solution to give solutions of different concentrations. Then, each concentration of the four compounds when S/N ≥ 3 and S/N ≥ 10 was detected 3 times by UPLC-FLD, and the average S/N was obtained.

#### 2.6.2. Linearity

Mobile phases A and B (31:69, *v*/*v*) were used to dilute the working standard solutions of ABZ, ABZ-SO_2_, ABZ-SO and ABZ-2NH_2_-SO_2_ to a series of concentrations (10.0, 20.0, 25.0, 50.0, 100.0, 200.0 and 400.0 μg/L for ABZ; 1.0, 10.0, 25.0, 50.0, 100.0, 200.0 and 400.0 μg/L for ABZ-SO_2_; 8.0, 10.0, 25.0, 50.0, 100.0, 200.0 and 400.0 μg/L for ABZ-SO; and 1.5, 10.0, 25.0, 50.0, 100.0, 200.0 and 400.0 μg/L for ABZ-2NH_2_-SO_2_), and these solutions were then analyzed 5 times by the optimized UPLC-FLD method. Calibration curves were prepared using the peak areas as the ordinate (Y) and the concentrations of the working solutions as the abscissa (X).

#### 2.6.3. Recovery and Precision

The recovery and precision were determined by analyzing blank muscle samples spiked with each compound in six replicates at the LOQ, 0.5 MRL, 1.0 MRL and 2.0 MRL levels. Recoveries were determined by comparing the chromatographic peak areas of extracted analytes from calibration curves of each compound. Precision, including intraday precision and interday precision, was evaluated by RSD. The intraday RSD was determined by analyzing blank muscle samples spiked with each compound in six replicates at the four concentration levels at different times on the same day, and the interday RSD was determined by analyzing blank muscle samples spiked with each compound in six replicates at the four concentration levels on different days.

## 3. Results and Discussion

### 3.1. Optimization of Chromatographic Conditions

#### 3.1.1. Selection of a Chromatographic Column

The chromatographic column plays a key role in successful analysis and is responsible for separation in an LC system. Based on previous reports on the detection of ABZ and its metabolites in animal-derived foods, the most commonly used chromatographic column was the C_18_ column [14,15]. In this study, HSS T3, CSH C_18_, BEH C_18_ and Shield RP18 columns were tested.

Initially, regardless of flow rate, column temperature and other related parameters, the chromatographic peaks of ABZ-SO and ABZ-2NH_2_-SO_2_ could not be separated well when the HSS T3 column or CSH C_18_ column was used for analysis, and overlapping peaks were generated. Therefore, separation could not be achieved using the HSS T3 or CSH C_18_ column. A series of sharp solvent peaks appeared when the Shield RP18 column was used, which resulted in the loss of the ABZ-SO peak. Finally, the ACQUITY UPLC^®^ BEH C_18_ chromatographic column was selected as the analytical column for this study. The peak shapes of the four target compounds were sharp and symmetric, optimal separation was achieved, and the peaks were not interfered with by peaks of impurities in the sample tissue. The analysis time was 6 min, and detection could be completed quickly under optimized UPLC-FLD conditions and using a BEH C_18_ column.

#### 3.1.2. Optimization of the Mobile Phase

The composition of the mobile phase has a substantial influence on the separation and peak shape of analytes. In relevant studies on the simultaneous detection of ABZ and its three metabolites in animal-derived foods, at present, the water (containing a small amount of organic acid)-acetonitrile [17,18,19] mobile phase system and the water (containing a small amount of formic acid)-methanol [21,22,23] mobile phase system are frequently used. In this study, when 69% water (containing 0.2% formic acid and 31% methanol was used as the mobile phase, the chromatogram showed large fluctuations at 4–6 min in the gradient elution procedure, which caused the ABZ peak to be lost. When 69% water (containing 0.2% formic acid) and 31% acetonitrile was selected as the mobile phase, high responses and sharp peak shapes were obtained for ABZ, ABZ-SO_2_, ABZ-SO and ABZ-2NH_2_-SO_2_. Other concentrations of formic acid were not tested because the effect of 0.2% formic acid was good. Moreover, the separation effects of six different ratios of water (containing 0.2% formic acid)-acetonitrile mobile phase systems (75:25, 73:27, 71:29, 69:31, 67:33, 65:35, *v*/*v*) were investigated. When the mobile phase ratio was 69:31, the baseline chromatogram was stable, there were no solvent peaks, and the separation effects of each target were optimal. However, the chromatographic peak had a small tail, which may be because ABZ and its three metabolites are weakly alkaline substances and are prone to ionization. An appropriate amount of triethylamine added to mobile phase B can inhibit ionization, relieve the phenomenon of chromatographic peak tailing, and yield good chromatographic peaks. The addition of different amounts (0.01%, 0.03%, 0.05%, 0.07% and 0.09%) of triethylamine to relieve the chromatographic peak tailing phenomenon was also examined. The peaks of each target analyte were symmetrical when the concentration of triethylamine was 0.05%. Thus, the study used 0.2% formic acid aqueous solution (containing 0.05% triethylamine)-acetonitrile (69:31, *v*/*v*) as the mobile phase for isocratic elution. Compared to the gradient elution procedures used in other methods, the isocratic elution procedure was simple and stable in this method.

#### 3.1.3. Optimization of Column Temperature and Flow Rate

Proper column temperature can promote the separation effect of the target analytes and eliminate the influence of environmental temperature changes. In this study, the chromatographic peak shapes of four target compounds were tested at different chromatographic column temperatures (25 °C, 30 °C, 31 °C, 33 °C, 35 °C, 37 °C and 40 °C). First, different column temperatures (25 °C, 30 °C, 35 °C and 40 °C) were compared. When the column temperature was under 30 °C, the retention times of the target analytes were delayed. When the column temperature was set to 40 °C or higher, the peak shape of the target analytes improved, but separation of the ABZ-SO and ABZ-2NH_2_-SO_2_ peaks was affected. Moreover, high temperatures caused irreversible damage to the column. The column temperatures of 31 °C, 33 °C, 35 °C, 37 °C and 39 °C were then compared. Ultimately, the retention times and separation of the target analytes were optimal when the column temperature was 35 °C. Therefore, a column temperature of 35 °C was selected in this study.

The column pressure of a chromatographic column increases with increasing flow rate. Decreasing the flow rate can prolong the analysis time. The effect of flow rate on the retention time of the target compounds was examined. Finally, the optimal flow rate was selected as 0.25 mL/min according to the separation and retention time of the target compounds.

### 3.2. Chromatograms and Determination of Detection Wavelengths

**Figure 2:**
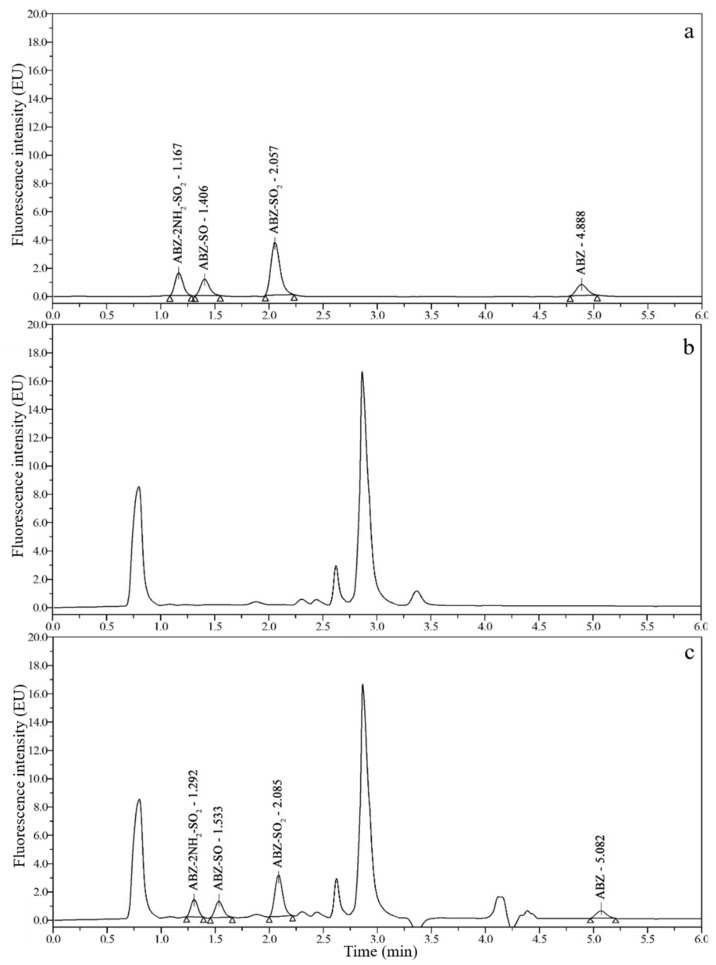
Chromatograms of 10 μg/kg ABZ-2NH_2_-SO_2_, 50 μg/kg ABZ-SO, 10 μg/kg ABZ-SO_2_ and 50 μg/kg ABZ standards (**a**); blank pig muscle (**b**); and blank pig muscle spiked with 10 μg/kg ABZ-2NH_2_-SO_2_, 50 μg/kg ABZ-SO, 10 μg/kg ABZ-SO_2_ and 50 μg/kg ABZ standards (**c**).

**Figure 3:**
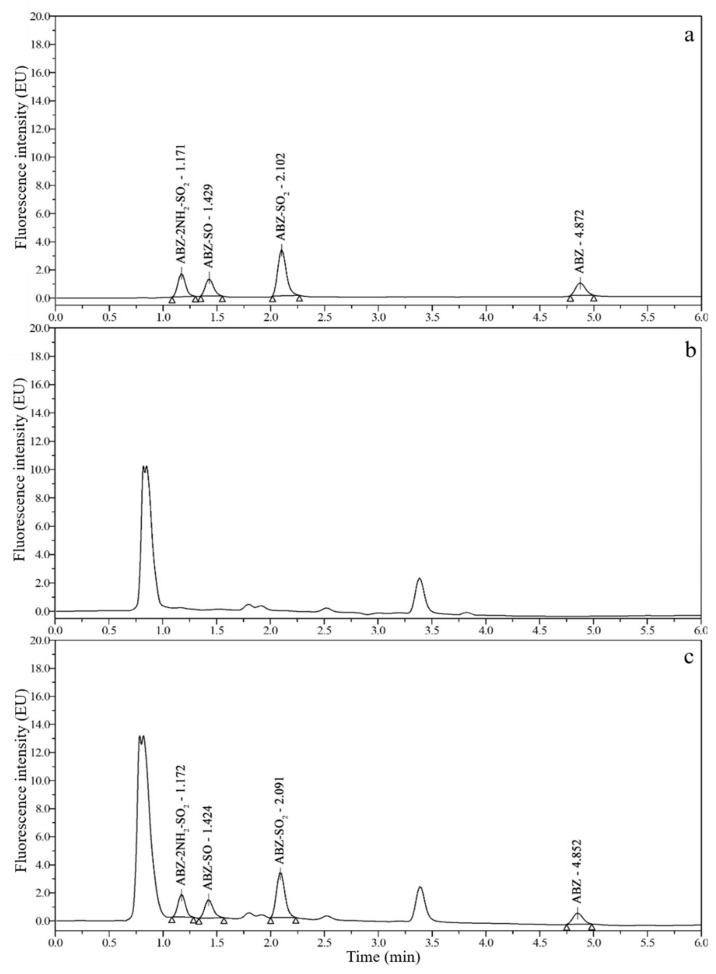
Chromatograms of 10 μg/kg ABZ-2NH_2_-SO_2_, 50 μg/kg ABZ-SO, 10 μg/kg ABZ-SO_2_ and 50 μg/kg ABZ standards (**a**); blank chicken muscle (**b**); and blank chicken muscle spiked with 10 μg/kg ABZ-2NH_2_-SO_2_, 50 μg/kg ABZ-SO, 10 μg/kg ABZ-SO_2_ and 50 μg/kg ABZ standards (**c**).

**Figure 4:**
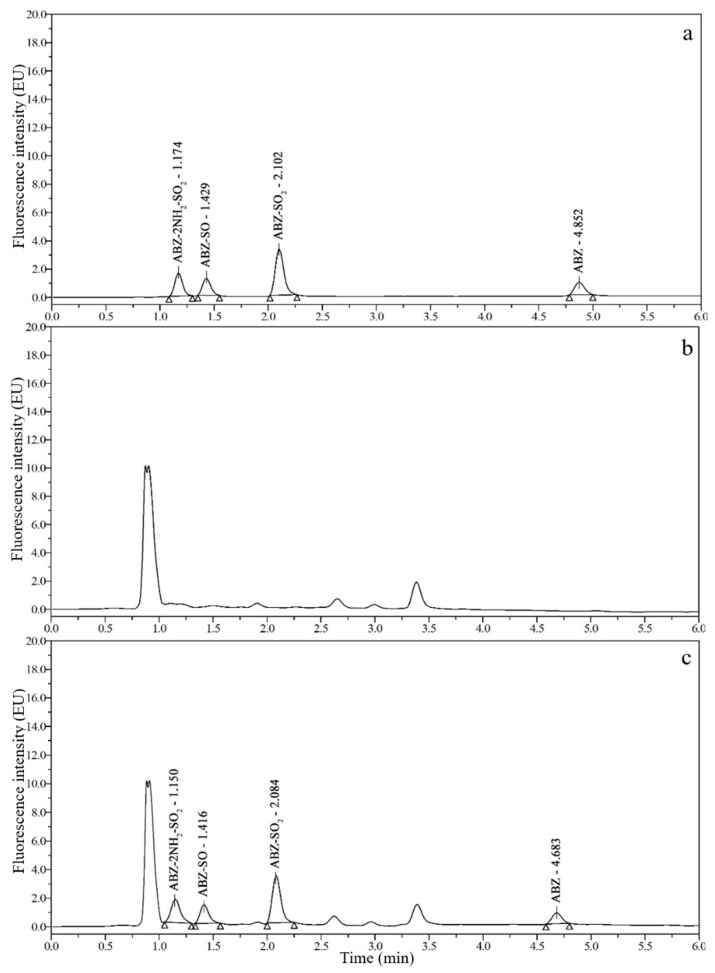
Chromatograms of 10 μg/kg ABZ-2NH_2_-SO_2_, 50 μg/kg ABZ-SO, 10 μg/kg ABZ-SO_2_ and 50 μg/kg ABZ standards (**a**); blank duck muscle (**b**); and blank duck muscle spiked with 10 μg/kg ABZ-2NH_2_-SO_2_, 50 μg/kg ABZ-SO, 10 μg/kg ABZ-SO_2_ and 50 μg/kg ABZ standards (**c**).

**Figure 5:**
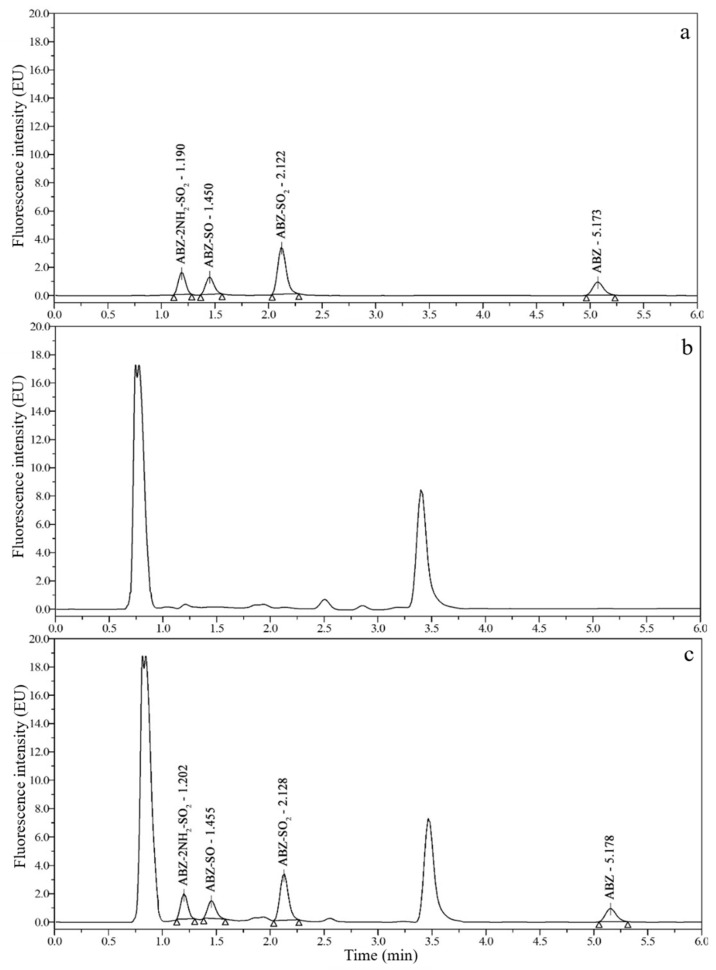
Chromatograms of 10 μg/kg ABZ-2NH_2_-SO_2_, 50 μg/kg ABZ-SO, 10 μg/kg ABZ-SO_2_ and 50 μg/kg ABZ standards (**a**); blank goose muscle (**b**); and blank goose muscle spiked with 10 μg/kg ABZ-2NH_2_-SO_2_, 50 μg/kg ABZ-SO, 10 μg/kg ABZ-SO_2_ and 50 μg/kg ABZ standards (**c**).

Good chromatograms (Figure 2, Figure 3, Figure 4 and Figure 5) were obtained by using the optimized UPLC-FLD conditions. As shown in Figure 2, Figure 3, Figure 4 and Figure 5, the retention times of ABZ, ABZ-SO_2_, ABZ-SO and ABZ-2NH_2_-SO_2_ were approximately 5.00, 2.10, 1.45, and 1.20 min, respectively. The peak shapes of the target compounds were well separated and did not overlap. The chromatographic peaks of the target compounds did not exhibit tailing and were not affected by impurity peaks.

Fluorescence detectors are highly selective detectors that are only suitable for detecting compounds containing fluorophores. Detection wavelengths (excitation wavelengths and emission wavelengths) are necessary parameters for fluorescence detection and can directly affect the sensitivity and selectivity of detection. According to some previous LC-FLD studies [12,13,14,15,16], the excitation wavelengths and emission wavelengths of ABZ and its three metabolites are 290 and 330 nm and 290 and 320 nm, respectively. In this study, the detection wavelengths of the four target compounds were scanned by a multimode plate reader, and the scan results showed that the excitation wavelengths and emission wavelengths of ABZ, ABZ-SO_2_, ABZ-SO and ABZ-2NH_2_-SO_2_ were 284.9 and 345.3 nm, 278.6 and 327.9 nm, 303.1 and 330.4 nm and 277.1 and 335.8 nm, respectively. According to the sensitivities and responses of each target, the excitation wavelengths and emission wavelengths of the four target compounds were chosen as 286 and 335 nm, respectively, after the different detection wavelengths of the four compounds were compared in this study.

### 3.3. Optimization of Sample Preparation

The choice of extractant for LLE was significant for the recovery of the four analytes from muscle samples and required both the ability to extract drugs and the ability to remove interfering substances in the sample matrix. ABZ and its three metabolites are all moderately polar molecules that are weakly alkaline. In previous studies, the most common solvent used to extract ABZ and its three metabolites in animal-derived foods was ethyl acetate [24,25] or acetonitrile [12,26]. In this research, the extraction effects of ethyl acetate and acetonitrile as extractants were compared. According to the recovery rate results, the extraction effect of acetonitrile was good. However, due to the high polarity of acetonitrile, more endogenous substances were extracted from pig and poultry muscle, which caused the chromatographic peaks of the target substances to be interfered with by the peaks of impurities during UPLC-FLD analysis. Ethyl acetate is low in toxicity and highly volatile, which could save time during concentration and improve detection efficiency. When ethyl acetate was used as the extractant, these target compounds could be effectively extracted from the sample matrix with a high recovery; additionally, the peaks of the target compounds were not interfered with by the peaks of impurities in the sample tissue during UPLC-FLD analysis. Therefore, ethyl acetate was used as the extractant in this study. Moreover, to improve the recovery rate, samples were extracted one, two, or three times, and the recoveries were compared. Two extractions could effectively increase the recovery rate compared with one extraction. Three extractions did not significantly improve the recovery rate and wasted more reagents. Finally, the samples were extracted two times to optimize the recovery rate and environmental protection.

Relevant literature has reported on different types of SPE cartridges and purification procedures to purify extracted samples, and MCX cartridges [18] and C_18_ cartridges [20,21] are commonly used. In this study, a comprehensive comparison of the purification effects of Waters Oasis MCX, ProElutAL-A acidic Al_2_O_3_, Cleanert S C_18_ and Waters PRIME HLB cartridges was performed. When Waters Oasis MCX, Cleanert S C_18_ and acidic Al_2_O_3_ cartridges were used for purification, the recoveries of ABZ and ABZ-2NH_2_-SO_2_ were significantly lower, and the purification effect was poor. The purification effect of the Waters PRIME HLB cartridges was good. There was no interference with the peaks of target compounds, and the recovery was high when Waters PRIME HLB cartridges were used. In addition, an oil-free vacuum pump and an antifogging glass vacuum tank were equipped during the SPE extraction process, which could avoid cross-contamination and improve the extraction efficiency.

A filter membrane was used to filter impurities and thus protect the chromatographic analysis system. A polyvinylidene fluoride (PVDF) hydrophobic syringe filter membrane, a hydrophilic polytetrafluoroethylene (PTFE) syringe filter membrane, an aqueous phase syringe filter membrane and an organic phase nylon syringe filter membrane were examined. The results showed that when the aqueous phase syringe filter membrane and PVDF syringe filter membrane were used, severe peak tailing occurred. The organic phase nylon syringe filter membrane had a large pore size and could not effectively filter out impurities. In contrast, the hydrophilic PTFE syringe filter membrane exhibited better performance. Not only did this filter provide clean extracts, but the recovery of the four compounds was also higher when a PTFE syringe filter membrane was used. Therefore, the hydrophilic PTFE syringe filter membrane (13 mm × 0.22 μm) was ultimately selected.

### 3.4. Method Comparison

To date, various analytical methods have been used for the simultaneous detection of ABZ and its three metabolites. However, to our knowledge, not all studies included ABZ and all three metabolites in the detection process, and there are no reports using a UPLC-FLD method for the simultaneous detection of ABZ and its three metabolites in animal-derived foods. Shaikh et al. [14] established an LC-FLD method for the determination of ABZ and its major metabolites in some fish muscles; the average recoveries were 67–94%, and the detection time was 20 min. Xu et al. [17] developed an LC-MS/MS method for determining mebendazole and its metabolites, ABZ and its metabolites, and levamisole in muscles of aquatic products. The recoveries of ABZ and its metabolites were 80.0–113.7%, with an RSD less than 10.0%, and the detection time was 10 min. Permana et al. [11] developed an HPLC-UV method determining ivermectin, ABZ, ABZ-SO_2_, ABZ-SO and doxycycline in rat plasma and organs. The recoveries of ABZ, ABZ-SO_2_ and ABZ-SO were 79.81–97.29%, with an RSD less than 11.14%, and the detection time was 20 min. The UPLC-FLD method in this study could simultaneously detect the residues of ABZ and its three metabolites in pig and poultry muscle using LLE combined with SPE technology to extract target analytes and purify the sample matrix. The recoveries of ABZ and its three metabolites from all samples were 80.37–98.39%, with an RSD less than 6.20%, the LODs and LOQs were 0.2–3.8 μg/kg and 1.0–10.9 μg/kg, respectively, and the detection time was completed within 6 min. Compared with other methods, the most significant advantages of the UPLC-FLD method were a shorter detection time (6 min), which greatly improves work efficiency, and the RSDs were better than those of the LC-FLD, LC-MS/MS and HPLC-UV methods. Moreover, the extraction recovery and sensitivity were comparable to those of most methods. Thus, the study provides a new and advanced technology for the efficient and rapid detection of ABZ and its three metabolites in animal products.

### 3.5. Validation Results of the Method

We did not find a suitable internal standard before the beginning of the research. Therefore, we chose the external standard method for this study.

#### 3.5.1. Sensitivity

The LODs and LOQs of ABZ and its three metabolites were examined in pig and poultry muscle in accordance with the method above. The determination results are listed in Table 1. As shown in Table 1, in pig and poultry muscle, the LOD of ABZ was 2.8–3.6 μg/kg, and the LOQ was 10.0–10.9 μg/kg; the LOD of ABZ-SO_2_ was 0.2–0.4 μg/kg, and the LOQ was 1.0–1.5 μg/kg; the LOD of ABZ-SO was 2.4–3.8 μg/kg, and the LOQ was 8.0–9.7 μg/kg; and the LOD of ABZ-2NH_2_-SO_2_ was 0.5–0.9 μg/kg, and the LOQ was 1.5–3.0 μg/kg.

#### 3.5.2. Linearity

The peak areas and concentration of each drug had a good linear relationship when the concentration ranges of ABZ, ABZ-SO_2_, ABZ-SO and ABZ-2NH_2_-SO_2_ were 10.0–400.0 μg/L, 1.0–400.0 μg/L, 8.0–400.0 μg/L and 1.5–400.0 μg/L, respectively. The linear ranges, linear regression equations and coefficients of determination (R^2^) of ABZ, ABZ-SO_2_, ABZ-SO and ABZ-2NH_2_-SO_2_ are shown in Table 2.

#### 3.5.3. Recovery and Precision

The results of the recovery and precision of all analytes are summarized in Table 3, Table 4, Table 5 and Table 6. As shown in Table 3, Table 4, Table 5 and Table 6, when the concentrations of ABZ and its three metabolites were added to pig and poultry muscle at LOQ (the smallest LOQ among the four animal matrices), 0.5 MRL, 1.0 MRL and 2.0 MRL, the recoveries of ABZ, ABZ-SO_2_, ABZ-SO and ABZ-2NH_2_-SO_2_ were 80.37–98.39%, 83.13–97.68%, 84.65–97.93% and 83.53–97.25%, respectively; the RSDs were 1.05–4.71%, 1.55–4.91%, 1.28–3.84%, and 0.84–4.65%, respectively; the intraday RSDs were 2.18–4.85%, 1.33–5.11%, 1.68–4.34% and 2.04–4.71%, respectively; and the interday RSDs were 2.23–6.20%, 2.10–5.10%, 2.72–5.03% and 2.50–6.29%, respectively.

### 3.6. Real Sample Analysis

To evaluate the feasibility and applicability of the new method, 40 samples each of chicken, duck, goose and pig muscle were analyzed by the developed method and were purchased from a local supermarket. Only two chicken muscle samples were found to contain ABZ-2NH_2_-SO_2_ residues (11.3 and 14.6 µg/kg), one pig muscle sample was found to contain ABZ-2NH_2_-SO_2_ residues (11.8 µg/kg), and none of the samples exceeded the MRL of 100 µg/kg (EU standard). Therefore, the novel UPLC-FLD method is reliable for application according to a real sample analysis.

## 4. Conclusions

A fast and effective LLE-SPE preparation method coupled with UPLC-FLD was established for detecting multiple residues of ABZ and its three metabolites in pig and poultry muscle. The extraction method exhibited high extraction efficiencies and recoveries, and the obtained recoveries of ABZ and its three metabolites in pig and poultry muscle were all greater than 80.37%. This LLE-SPE-UPLC-FLD method provides a new method for the simultaneous detection of ABZ and its three metabolites in pig and poultry muscle, and this method was found to have relatively high sensitivity and precision and a short detection time. Moreover, the newly developed method was successfully applied to the analysis of real samples, which proved the applicability of this method.

## Figures and Tables

**Table 1:** LODs and LOQs of ABZ, ABZ-SO_2_, ABZ-SO and ABZ-2NH_2_-SO_2_ in pig and poultry muscle.

Analyte	Animal Matrix	LOD (μg/kg)	LOQ (μg/kg)
ABZ	Pig muscle	2.8	10.0
	Chicken muscle	3.2	10.7
	Duck muscle	3.1	10.0
	Goose muscle	3.6	10.9
ABZ-SO_2_	Pig muscle	0.4	1.5
	Chicken muscle	0.3	1.0
	Duck muscle	0.2	1.0
	Goose muscle	0.2	1.0
ABZ-SO	Pig muscle	3.8	9.7
	Chicken muscle	3.0	8.3
	Duck muscle	2.4	8.0
	Goose muscle	2.5	8.5
ABZ-2NH_2_-SO_2_	Pig muscle	0.9	3.0
	Chicken muscle	0.6	1.8
	Duck muscle	0.5	1.5
	Goose muscle	0.5	1.9

**Table 2:** The linearity range, linear regression equation and determination coefficient of ABZ, ABZ-SO_2_, ABZ-SO and ABZ-2NH_2_-SO_2_ (n = 5).

Analyte	Linearity Range (μg/L)	Linear Regression Equation	Determination Coefficient (R^2^)
ABZ	10.0–400.0	y = 1355.7x + 11,560	0.9995
ABZ-SO_2_	1.0–400.0	y = 32,757x + 61,988	0.9994
ABZ-SO	8.0–400.0	y = 2498.1x + 14,519	0.9991
ABZ-2NH_2_-SO_2_	1.5–400.0	y = 13,573x + 32,379	0.9995

**Table 3:** Recovery and precision of ABZ, ABZ-SO_2_, ABZ-SO and ABZ-2NH_2_-SO_2_ added to blank pig muscle (n = 6).

Analyte	Adding Level (μg/kg)	Recovery (%)	RSD (%)	Intraday RSD (%)	Interday RSD (%)
ABZ	10.0	80.37 ± 1.87	2.33	4.67	5.77
	50.0	85.55 ± 2.28	2.67	3.42	6.20
	100.0 ^α^	89.67 ± 2.12	2.36	3.17	5.74
	200.0	92.07 ± 0.97	1.05	2.54	3.56
ABZ-SO_2_	1.5	91.69 ± 3.42	3.73	4.53	3.54
	50.0	93.54 ± 3.27	3.49	2.97	4.35
	100.0 ^α^	95.44 ± 2.14	2.24	2.15	3.87
	200.0	97.08 ± 2.02	2.08	3.37	4.84
ABZ-SO	9.7	84.65 ± 2.97	3.51	4.34	4.77
	50.0	91.79 ± 1.73	1.89	3.04	5.03
	100.0 ^α^	92.49 ± 3.43	3.70	3.17	3.64
	200.0	97.93 ± 2.73	2.79	2.70	2.93
ABZ-2NH_2_-SO_2_	3.0	84.56 ± 3.93	4.65	4.71	5.03
	50.0	90.48 ± 2.16	2.39	2.52	6.29
	100.0 ^α^	96.58 ± 1.36	1.41	3.77	4.79
	200.0	97.18 ± 2.23	2.30	3.10	4.35

Note: “α”. Maximum residue limits.

**Table 4:** Recovery and precision of ABZ, ABZ-SO_2_, ABZ-SO and ABZ-2NH_2_-SO_2_ added to blank chicken muscle (n = 6).

Analyte	Adding Level (μg/kg)	Recovery (%)	RSD (%)	Intraday RSD (%)	Interday RSD (%)
ABZ	10.7	88.77 ± 1.89	2.13	2.18	2.63
	50.0	89.61 ± 2.73	3.05	3.24	3.81
	100.0 ^α^	94.77 ± 1.15	1.22	2.85	3.47
	200.0	98.39 ± 1.50	1.53	2.88	2.23
ABZ-SO_2_	1.0	83.13 ± 3.94	4.74	5.11	4.91
	50.0	89.36 ± 1.60	1.79	2.84	2.10
	100.0 ^α^	94.01 ± 3.38	3.60	3.62	3.14
	200.0	97.12 ± 2.76	2.84	2.89	2.85
ABZ-SO	8.3	88.08 ± 3.38	3.84	4.10	4.77
	50.0	89.65 ± 3.13	3.49	3.72	4.11
	100.0 ^α^	94.39 ± 2.73	2.90	3.00	3.27
	200.0	94.66 ± 2.68	2.83	2.88	3.04
ABZ-2NH_2_-SO_2_	1.8	83.53 ± 2.68	3.20	3.57	3.98
	50.0	88.88 ± 3.35	3.77	4.25	4.65
	100.0 ^α^	94.07 ± 1.28	1.36	2.08	2.95
	200.0	95.34 ± 1.79	1.87	2.77	3.36

Note: “α”. Maximum residue limits.

**Table 5:** Recovery and precision of ABZ, ABZ-SO_2_, ABZ-SO and ABZ-2NH_2_-SO_2_ added to blank duck muscle (n = 6).

Analyte	Adding Level (μg/kg)	Recovery (%)	RSD (%)	Intraday RSD (%)	Interday RSD (%)
ABZ	10.0	89.00 ± 2.19	2.46	3.00	3.38
	50.0	91.50 ± 2.91	3.18	3.64	3.49
	100.0 ^α^	89.60 ± 1.64	1.83	2.37	2.79
	200.0	97.51 ± 1.87	1.92	2.34	2.69
ABZ-SO_2_	1.0	88.14 ± 4.33	4.91	4.64	5.10
	50.0	90.58 ± 3.23	3.57	4.03	4.45
	100.0 ^α^	93.44 ± 2.11	2.26	2.62	2.97
	200.0	95.55 ± 2.00	2.09	2.71	2.84
ABZ-SO	8.0	87.37 ± 2.99	3.43	3.58	4.02
	50.0	93.77 ± 2.78	2.96	3.12	2.97
	100.0 ^α^	91.05 ± 3.36	3.69	3.88	4.38
	200.0	96.20 ± 2.68	2.79	2.90	3.39
ABZ-2NH_2_-SO_2_	1.5	90.84 ± 3.60	3.96	3.98	3.95
	50.0	90.21 ± 2.13	2.36	2.66	3.04
	100.0 ^α^	95.74 ± 1.35	1.41	2.04	2.90
	200.0	96.09 ± 2.20	2.29	2.27	2.50

Note: “α”. Maximum residue limits.

**Table 6:** Recovery and precision of ABZ, ABZ-SO_2_, ABZ-SO and ABZ-2NH_2_-SO_2_ added to blank goose muscle (n = 6).

Analyte	Adding Level (μg/kg)	Recovery (%)	RSD (%)	Intraday RSD (%)	Interday RSD (%)
ABZ	10.9	84.09 ± 3.96	4.71	4.85	5.36
	50.0	88.72 ± 2.21	2.49	2.35	2.47
	100.0 ^α^	90.36 ± 2.09	2.32	2.40	3.92
	200.0	95.09 ± 3.62	3.81	4.27	4.71
ABZ-SO_2_	1.0	89.11 ± 1.59	1.79	1.33	3.80
	50.0	93.84 ± 2.17	2.31	2.34	2.56
	100.0 ^α^	95.19 ± 2.63	2.76	2.62	2.92
	200.0	97.68 ± 1.51	1.55	1.67	3.50
ABZ-SO	8.5	87.98 ± 3.32	3.78	3.89	4.07
	50.0	88.37 ± 1.13	1.28	1.68	3.40
	100.0 ^α^	92.49 ± 2.63	2.85	3.76	3.42
	200.0	97.03 ± 2.15	2.21	2.11	2.72
ABZ-2NH_2_-SO_2_	1.9	91.83 ± 3.24	3.53	3.56	3.63
	50.0	92.03 ± 2.70	2.94	2.91	3.06
	100.0 ^α^	94.26 ± 2.30	2.44	2.77	3.11
	200.0	97.25 ± 0.81	0.84	2.47	3.41

Note: “α”. Maximum residue limits.
